# Interrelation between the lipid accumulation product index and diabetic kidney disease in patients with type 2 diabetes mellitus

**DOI:** 10.3389/fendo.2023.1224889

**Published:** 2023-08-14

**Authors:** Min Tang, Shuangshuang Yao, Han Cao, Xiaohui Wei, Qin Zhen, Yijiong Tan, Fang Liu, Yufan Wang, Yongde Peng, Nengguang Fan

**Affiliations:** ^1^Department of Endocrinology and Metabolism, Shanghai General Hospital, Shanghai Jiao Tong University School of Medicine, Shanghai, China; ^2^Department of Endocrinology and Metabolism, Shanghai General Hospital of Nanjing Medical University, Shanghai, China; ^3^Department of Endocrinology, Songjiang District Central Hospital, Shanghai, China; ^4^Department of Clinical Pharmacy, Shanghai General Hospital, Shanghai Jiao Tong University School of Medicine, Shanghai, China

**Keywords:** lipid accumulation product, abdominal obesity, restricted cubic spline, type 2 diabetes mellitus, diabetic kidney disease

## Abstract

**Objective:**

The purpose of this study was to determine the relation between the lipid accumulation product index (LAPI) and diabetic kidney disease (DKD) in patients with type 2 diabetes mellitus (T2DM).

**Methods:**

Herein, 931 patients were enrolled and their data were collected. Then the interrelation between LAPI and DKD was assessed using multivariate logistic regression analyses (LRAs) and by a restricted cubic spline (RCS).

**Results:**

In total, 931 participants (352 females and 579 males) aged 55 years on average were included in the study. After adjusting for several confounders, the odds ratio for DKD was increased evidently in the third LAPI tertile compared with that in the first LAPI tertile. In addition, the RCS revealed a positive interrelation between LAPI and DKD. In the subgroup analyses, age, sex, hyperlipidemia, hypertension, and HbA1c did not significantly interact with LAPI.

**Conclusions:**

LAPI was higher in the DKD group than in the no-DKD group, and LAPI is positively linked with DKD, which may have potential value to diagnose DKD in clinical practice.

## Introduction

In 2021, about 10.5% of the global population aged 20–79 years suffered from diabetes. This percentage is expected to rise to approximately 12.2% in 2045 (1). The proportion of diabetes patients with type 2 diabetes mellitus (T2DM) is estimated to be 90% (2). Diabetes can lead to disability and mortality, which largely result from diabetic kidney disease (DKD) (3). DKD has become a major cause of end-stage renal disease (ESRD), and the incidence of DKD increases as the global prevalence of diabetes rises (1). Of all ESRD cases worldwide, 30–50% are estimated to be caused by DKD (4). Moreover, DKD increases the risk of cardiovascular and cerebrovascular diseases (4). Early surveillance and treatment of DKD are critical for reducing the burden on the healthcare system. Therefore, an effective tool for screening patients at a high risk of DKD is necessary.

Accumulating evidence has revealed that abdominal obesity (AO) is related to diabetes and its complications (5, 6). Moreover, it has been unveiled that relative to the total amount of adipose tissue, the distribution of adipose tissue is more important in the progression of vascular complications (7, 8). Some examinations, such as computed tomography and magnetic resonance imaging, can precisely detect abdominal adiposity. However, these examinations cannot be widely used because of their high cost and inconvenience. Therefore, indicators for AO, such as the body mass index (BMI), waist circumference (WC), waist-to-hip ratio (WHR) (9), waist-to-height ratio (WHtR) (10), the lipid accumulation product index (LAPI) (11), the visceral adiposity index (VAI) (12), and the Chinese visceral adiposity index (CVAI) (13), have been employed to assess abdominal adiposity. LAPI is a new marker of central lipid accumulation based on serum triglycerides and waist circumference, which was proposed to assess the lipid overaccumulation (11). Previous study indicated that LAPI had the ability to predict the risk of metabolic syndrome and T2DM (14, 15), cardiovascular events (16), non-alcoholic fatty liver disease (17). Moreover, LAPI may be an accurate marker of insulin resistance (18). However, little is known about the interrelation between LAPI and DKD.

Therefore, we conducted a cross-sectional study to decipher the relation between LAPI and DKD.

## Methods

### Study design

This study was a cross-sectional study. This study was approved by the Ethics Committee of the hospital. Informed consent was provided by all subjects before participating.

### Study subjects

The participants between April 2017 and September 2021 were obtained from the electronic medical database of the National Metabolic Management Center (MMC) of Shanghai General Hospital (Songjiang District), which was established to conveniently and precisely diagnose and treat metabolic diseases (19). The inclusion criteria were as follows: patients met the World Health Organization’s 1999 criteria for diagnosis of T2DM (20) and patients were aged >18 years. The exclusion criteria were as follows: pregnancy, malignant tumor, chronic nephritis, and missing data. After screening, a total of 931 individuals were enrolled in this study.

### Patient data collection

Data including name, sex, age, educational level, coexisting diseases, and medical therapy were collected. A variety of anthropometric measurements were taken, including WC, hip circumference (HC), height, weight, blood pressure (BP), and heart rate. WC and HC were evaluated by trained staff in accordance with standard protocols. Biochemical data were collected from the MMC, including leukocyte counts, hemoglobin levels, and high-sensitivity C-reactive protein (hs-CRP) levels.

### Definition of variables

A diagnosis of hypertension was made when systolic BP (SBP) ≥ 140 mmHg and/or diastolic BP (DBP) ≥ 90 mmHg during repeated examinations (21), patients used antihypertensive drugs, or patients had a history of hypertension. A diagnosis of hyperlipidemia was made when total cholesterol (TC) ≥ 5.2 mM, triglycerides (TG) ≥1.7 mM, patients used lipid-lowering medications, or patients had a prior history of hyperlipidemia. The definition of current smokers was those who were smoking cigarettes currently, and the definition of current drinkers was those who drank alcohol currently. Educational level was with two categories of under high school and high school or above.

BMI was calculated as per the following formulas: BMI = weight [kg]/height [m^2^]. The formulas to calculate LAPI was previously published (12, 22): males: LAPI = [WC(cm)−65] × TG(mmol/L); females: LAPI = [WC(cm)−58] × TG(mmol/L). Estimated glomerular filtration rate (eGFR) scores were computed with reference to a previous study (23). We calculated the albumin (ALB)-to-creatinine ratio (ACR) based on the following formula: ACR = ALB/creatinine. The diagnosis of DKD was made in accord with a previous study (24): ACR was higher than 30 mg/g or eGFR<60 mL/min per 1.73 m^2^.

### Statistical analyses

Numbers or medians (interquartile range) are used to present data. Continuous variables with skewed distributions were parsed by use of the Mann–Whitney U test, and an analysis of categorical variables was conducted for pairwise comparison using the χ2 test. *P* (two-sided) < 0.05 was indicative of statistical significance. SPSS 13.0 and R-4.1.3 were employed for statistical processing.

LAPI was divided into tertiles; the first tertile of LAPI was the lowest tertile, and the third tertile was the highest tertile. How LAPI is linked with DKD was parsed via multivariate logistic regression analyses (LRAs). Model 1 was adjusted for sex and age, Model 2 was further adjusted for DBP, SBP, educational level, and current drinking status, Model 3 was further adjusted for the duration of diabetes and hypertension, and Model 4 was further adjusted for fasting plasma glucose (FPG), leukocyte, ALB, hs-CRP, and glycated hemoglobin (HbA1c) levels. The interrelation between LAPI and DKD was examined by use of a restricted cubic spline (RCS) with four knots at the 5th, 35th, 65th, and 95th percentiles.

Additionally, by multivariate LRAs, we examined the association of LAPI with DKD based on age, sex, hyperlipidemia, hypertension, and HbA1c in different subgroups and computed the odds ratio (OR) and 95% confidence interval (CI). Moreover, we evaluated how the aforementioned subgroup variables interact with LAPI.

## Results

### Baseline characteristics of the study subjects

In total, 931 participants (352 females and 579 males) aged 55 years on average were enrolled. Of these participants, 327 had DKD and 604 did not. The two groups displayed significant differences in clinical data such as age, sex, educational level, hypertension, current drinker, SBP, DBP, height, BMI, WC, duration of diabetes, FPG, HbA1c, hemoglobin, leukocytes, gamma-glutamyl transferase, ALB, TG, TC, hs-CRP, ACR, and eGFR. Clinical data are illustrated in **Table 1**.

**Table 1:** Characteristics of Patients.

Variables	No DKD (n = 604; 64.88%)	DKD (n = 327; 35.12%)	*P* value
Age (years)	55 (42-63)	55 (47-65)	0.011
Diastolic blood pressure (mmHg)	75 (67-80)	78 (69-84)	<0.001
Systolic blood pressure (mmHg)	120 (113-130)	128 (120-140)	<0.001
Heart rate (beats per minute)	78 (76-86)	78 (75-88)	0.155
Height (cm)	167 (160-173)	164 (158-172)	0.022
Weight (kg)	69.2 (61.0-78.0)	69.8 (63.4-80.0)	0.177
BMI (kg/m^2^)	24.9 (23.0-27.3)	25.8 (23.6-28.4)	0.001
WC (cm)	92 (86-96)	93 (88-100)	0.004
HC (cm)	96 (93-100)	96 (93-101)	0.116
LAPI	45.92 (29.25-72.94)	60.52 (39.90-90.65)	<0.001
Duration of diabetes (months)	61 (1-144)	97 (12-171)	0.001
FPG (mmol/L)	6.9 (5.6-8.5)	7.7 (6.0-9.5)	<0.001
HbA1c (%)	8.6 (7.2-10.6)	9.3 (7.5-11.0)	0.003
Hemoglobin (g/L)	145 (132-154)	137 (124-148)	<0.001
Leukocyte (×10^9^/L)	5.99 (5.10-7.11)	6.50 (5.45-7.69)	<0.001
ALT (IU/L)	20 (14-34)	20 (14-30)	0.184
AST (IU/L)	18 (15-24)	18 (15-25)	0.500
GGT (IU/L)	24 (16-39)	27 (18-41)	0.004
Albumin (g/L)	43.2 (40.8-45.5)	42.3 (39.2-45.0)	<0.001
Blood urea nitrogen (mmol/L)	5.20 (4.12-6.84)	5.74 (4.47-7.76)	0.001
Creatinine (μmol/L)	58.35 (47.70-67.63)	58.70 (45.00-79.80)	0.204
Triglyceride (mmol/L)	1.58 (1.14-2.25)	1.87 (1.40-2.76)	<0.001
Total cholesterol (mmol/L)	4.56 (3.73-5.23)	4.71 (3.87-5.50)	0.019
HDL-C (mmol/L)	0.91 (0.79-1.08)	0.90 (0.78-1.05)	0.622
LDL-C (mmol/L)	2.61 (1.91-3.17)	2.60 (1.96-3.34)	0.450
Hs-CRP (mg/L)	1.2 (0.5-2.6)	1.9 (0.9-4.3)	<0.001
ACR (μg/mg)	13.16 (8.71-19.18)	71.83 (41.90-244.71)	<0.001
eGFR (mL/min per 1.73 m^2^)	110.90 (100.81-123.03)	105.62 (88.15-119.26)	<0.001
Sex(male/female)	400/204	179/148	0.001
Educational level (under high school/high school or above)	300/304	140/187	0.045
Hypertension(no/yes)	367/237	124/203	<0.001
Hyperlipidemia(no/yes)	455/149	233/94	0.176
Current smoker(no/yes)	415/189	239/88	0.163
Current drinker(no/yes)	394/210	237/90	0.024

Data are presented N or median (interquartile range). Continuous variables with skewed distribution used Mann-Whitney U test and categorical variables used chi-squared test for comparing the baseline characteristics of patients with diabetic kidney disease and without diabetic kidney disease. BMI, body mass index; WC, waist circumference; HC, hip circumference; LAPI, lipid accumulation product index; FPG, fasting plasma glucose; HbA1c, glycated hemoglobin; ALT, alanine transaminase; AST, aspartate aminotransferase; GGT, gamma -glutamyl transferase; HDL-C - high-density lipoprotein cholesterol; LDL-C - low-density lipoprotein cholesterol; Hs-CRP, high-sensitivity C-reactive protein; ACR, albumin to creatinine ratio; eGFR, estimated glomerular infiltration rate; DKD, diabetic kidney disease.

### Association between LAPI and DKD

**Figure 1:**
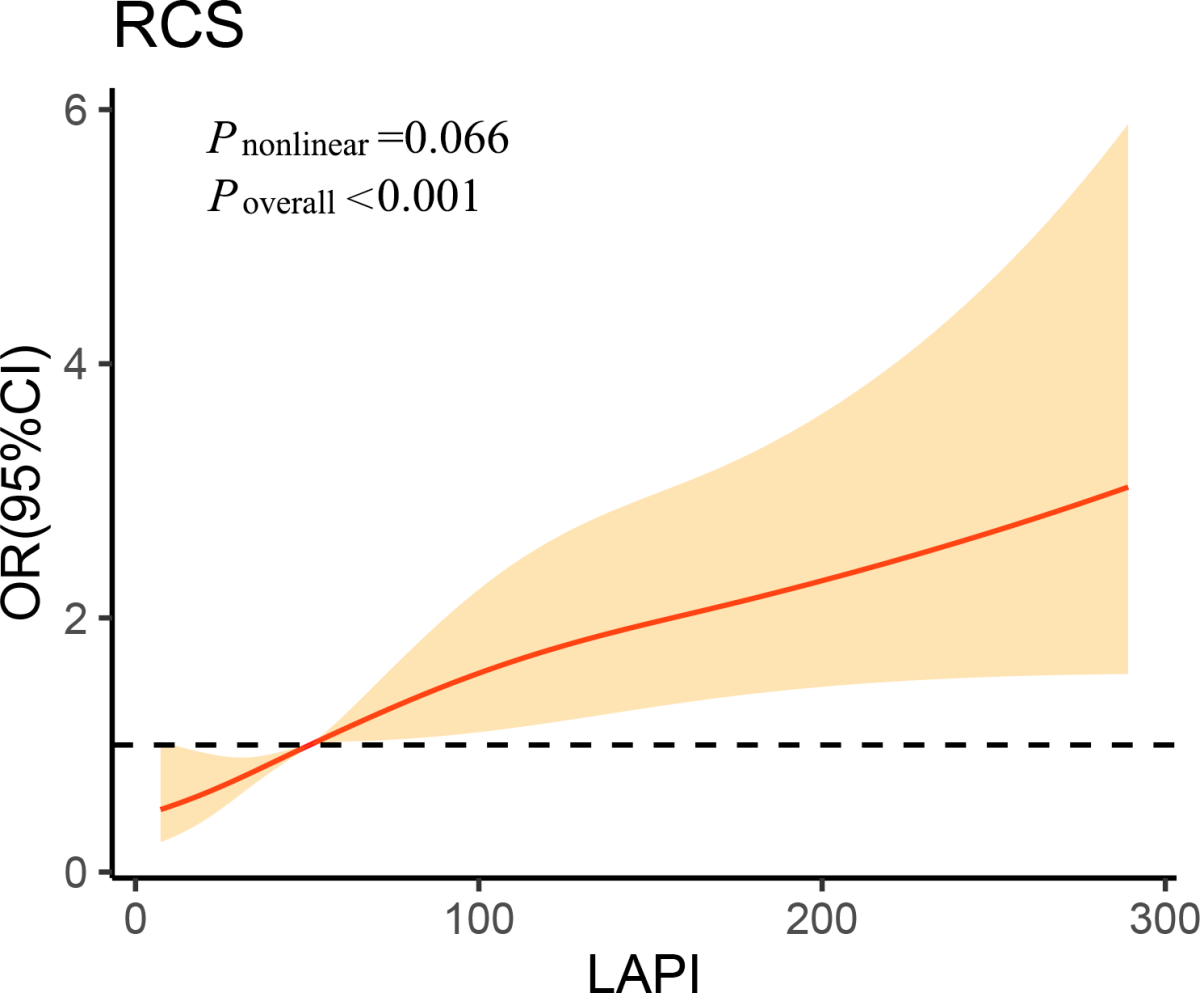
Association of lipid accumulation product index on a continuous scale with diabetic kidney disease in study participants. The solid line represents the odds ratio and the shad area represents the 95% confidence interval. Model was adjusted for age, diastolic blood pressure, systolic blood pressure, educational level, current drinker, duration of diabetes, hypertension, fasting plasma glucose, glycated hemoglobin, leukocyte, albumin and high-sensitivity C-reactive protein. OR, odds ratio; CI, confidence interval; RCS, restricted cubic spline; LAPI, lipid accumulation product index.

As demonstrated by multivariate LRAs, after adjusting for several confounding factors, elevated LAPI was associated with a higher likelihood of DKD (**Table 2**). Subsequent to the adjustment of the multivariate regression model for age, sex, DBP, SBP, educational level, current drinker, duration of diabetes, hypertension, FPG, HbA1c, leukocytes, ALB, and hs-CRP, higher OR values for DKD were found in the third LAPI tertile than in the first LAPI tertile (*P* < 0.001), and the OR (95% CI) for DKD was 2.135 (1.438–3.171) in the third tertile of LAPI compared to that in the first tertile. Additionally, RCS was performed to identify the relationship of LAPI with DKD. Consistently, the RCS curve demonstrated a positive relationship between LAPI and DKD in the study participants (**Figure 1**).

**Table 2:** Relations of lipid accumulation product index with diabetic kidney disease in patients with type 2 diabetes mellitus.

LAPI				
	T1 (N=311)	T2 (N=310)	T3 (N=310)	*P* value for trend
Model 1 - OR (95% CI)	Ref.	1.667 (1.174–2.368)	2.587 (1.819–3.679)	<0.001
Model 2 - OR (95% CI)	Ref.	1.558 (1.084–2.241)	2.407 (1.667–3.476)	<0.001
Model 3 - OR (95% CI)	Ref.	1.431 (0.988–2.074)	2.168 (1.490–3.156)	<0.001
Model 4 - OR (95% CI)	Ref.	1.391 (0.944–2.051)	2.135 (1.438–3.171)	<0.001

Model 1 was adjusted for sex, age. Model 2 was further adjusted for diastolic blood pressure, systolic blood pressure, educational level, current drinker. Model 3 was further adjusted for duration of diabetes, hypertension. Model 4 was further adjusted for fasting plasma glucose, glycated hemoglobin, leukocyte, albumin, high-sensitivity C-reactive protein. The quartile ranges of T1, T2 and T3 of LAPI were <38.08, 38.08-67.65,>67.65, respectively. T1 is the reference group. Multivariate logistic regression analyses were performed to estimate the ORs and corresponding 95% CIs for diabetic kidney disease. Abbreviations: T: tertile, OR: odds ratio, CI: confidence interval, LAPI: lipid accumulation product index.

### Subgroup analyses

**Figure 2:**
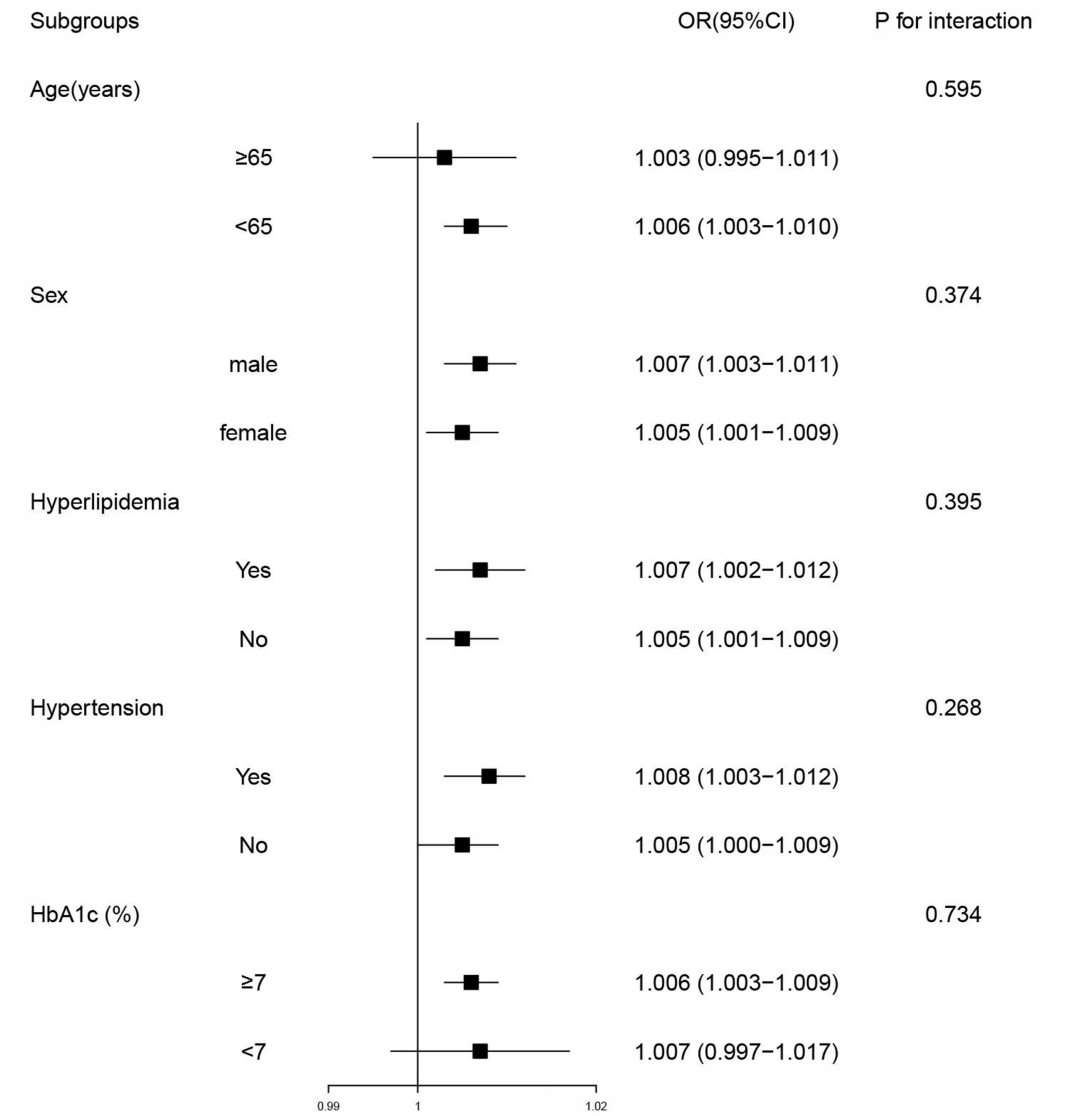
Subgroup analyses of associations between lipid accumulation product index and diabetic kidney disease. Model was adjusted for age, sex, diastolic blood pressure, systolic blood pressure, educational level, current drinker, duration of diabetes, hypertension, fasting plasma glucose, glycated hemoglobin, leukocyte, albumin and high-sensitivity C-reactive protein. Subgroup variable was excluded from the model. OR, odds ratio; CI, confidence interval; HbA1c, glycated hemoglobin.

To further investigate the association of LAPI with DKD in different populations, the participants were grouped by age (<65 or ≥65 years), hyperlipidemia (N or Y), sex (female or male), HbA1c (<7% or ≥7%), and hypertension (N or Y). In most categories, LAPI was positively associated with DKD according to the subgroup analyses. Age, sex, hyperlipidemia, hypertension, and HbA1c did not significantly interact with LAPI (**Figure 2**).

## Discussion

In conclusion, LAPI was higher in the DKD group than in the no-DKD group, and a positive relationship between LAPI and DKD was found after adjusting several potential confounders. Therefore, LAPI may have potential value to diagnose DKD in T2DM patients in clinical practice.

AO has been shown to be associated with a high risk of DKD in T2DM (25–27). A population-based cohort study has reported longitudinal associations of VAI and CVAI with DKD and suggested that VAI is independently related to the incidence of DKD (28). LAPI was calculated using TG and WC, which are novel indicators of central lipid accumulation (11). Accumulating evidence indicates that patients with diabetes display a higher LAPI level (14, 29). One study reported that the ability of single LAPI to predict CKD was better than that of VAI (30). Another study unveiled that several indicators pertaining to obesity, including BMI, WHR, WHtR, LAPI, and VAI, show relevance to kidney disease in late-stage T2DM patients (31). Similar results were obtained in the present study, we found that LAPI, one indicator for AO, was positively associated with DKD, which provides new scientific evidence for further study.

A previous study indicated that plasma TG levels are associated with reduced eGFR in new-onset cases of T2DM (32), and a high TG level contributes to DKD progression (33). These results are consistent with our conclusion that LAPI is positively associated with DKD. It is well acknowledged that oxidative stress and inflammation are common causes of DKD (34). Lipids accumulated in the kidney may induce oxidative stress or promote the release of proinflammatory cytokines, resulting in glomerular damage, glomerulosclerosis, and interstitial fibrosis (35, 36). Dysfunction of lipid metabolism in DKD could lead to podocyte damage, thus promoting DKD progression, which results in the disruption of mitochondrial function and lipid metabolism (37).

LAPI is recognized as a new indicator of accumulated visceral adipose tissue (38). Several studies reported that LAPI can be used to evaluate the risk of metabolic syndrome, diabetes, and cardiovascular disease (11, 14, 39). A higher LAPI in patients with T2DM was associated with increased oxidative stress, insulin resistance, and inflammation (40). The use of LAPI to assess visceral adipose tissue is characterized by higher lipolytic and proinflammatory activity (41). LAPI can be calculated easily, quickly and cheaply, which makes it suitable for use in clinical practice and in a large population (42). Our study revealed that LAPI was associated with DKD, so LAPI may be a useful marker of DKD in T2DM patients.

The strengths of the current study deserved to be mentioned. The study found the dose-response relationship between LAPI and DKD, and LAPI was evidently linked with DKD, which may be a potential index to identify DKD in clinical practice. There were a number of limitations to this study. First, it was cross-sectional study; longitudinal associations between LAPI and DKD need to be examined in future studies. Second, this study had a small sample size. In order to verify these results, further studies with larger samples are needed. Third, the insulin resistance index and the effects of lipid-lowering drugs were not analyzed in our study. Finally, further investigations of different ethnic groups are essential to examine the association between LAPI and DKD.

## Conclusion

In conclusion, LAPI was significantly and positively associated with DKD in T2DM patients. Therefore, LAPI may be a potential marker for identifying DKD in T2DM. And longitudinal associations between LAPI and DKD need to be examined in further study.

## Data availability statement

The original contributions presented in the study are included in the article/supplementary material. Further inquiries can be directed to the corresponding authors.

## Ethics statement

The studies involving humans were approved by the Ethics Committee of Shanghai General Hospital, Shanghai Jiao Tong University School of Medicine. The studies were conducted in accordance with the local legislation and institutional requirements. The participants provided their written informed consent to participate in this study.

## Author contributions

NF and YP designed this study. MT and SY collected and analyzed the study data. All authors wrote and reviewed the manuscript of the study. All authors approved the final manuscript.
